# Optimal PSF Estimation for Simple Optical System Using a Wide-Band Sensor Based on PSF Measurement

**DOI:** 10.3390/s18103552

**Published:** 2018-10-19

**Authors:** Yunda Zheng, Wei Huang, Yun Pan, Mingfei Xu

**Affiliations:** 1 State Key Laboratory of Applied Optics, Changchun Institute of Optics, Fine Mechanics and Physics, Chinese Academy of Sciences, Changchun 130033, China; yundazheng@foxmail.com (Y.Z.); xumf@ciomp.ac.cn (M.X.); 2 University of Chinese Academy of Sciences, Beijing 100049, China; panyun15@mails.ucas.edu.cn

**Keywords:** imaging sensors, point spread function, image restoration, deconvolution, simple optical system

## Abstract

Simple optical system imaging is a method to simplify optical systems by removing aberrations using image deconvolution. The point spread function (PSF) used in deconvolution is an important factor that affects the image quality. However, it is difficult to obtain optimal PSFs. The blind estimation of PSFs relies heavily on the information in the image. Measured PSFs are often misused because real sensors are wide-band. We present an optimal PSF estimation method based on PSF measurements. Narrow-band PSF measurements at a single depth are used to calibrate the optical system; these enable the simulation of real lenses. Then, we simulate PSFs in the wavelength pass range of each color channel all over the field. The optimal PSFs are computed according to these simulated PSFs. The results indicated that the use of the optimal PSFs significantly reduces the artifacts caused by misuse of PSFs, and enhances the image quality.

## 1. Introduction

Optical aberration is an important factor in image-quality degradation. Designers of modern optical imaging systems must use lenses of different glass material to obtain aberration corrected and balanced [1], which makes optical systems cumbersome and expensive. Fortunately, spatially varying deconvolution can reduce many aberrations [2]. The use of deconvolution methods in image post-processing enables the constraints in optical system design to be relaxed, and the optical system can be simplified [3]. Deconvolution is the key part of this strategy, and the point spread function (PSF) is an important factor that affects the deconvolution result [4,5]. Once the PSF is obtained inaccurately, the restored image is likely to have severe artifacts.

**Figure 1:**
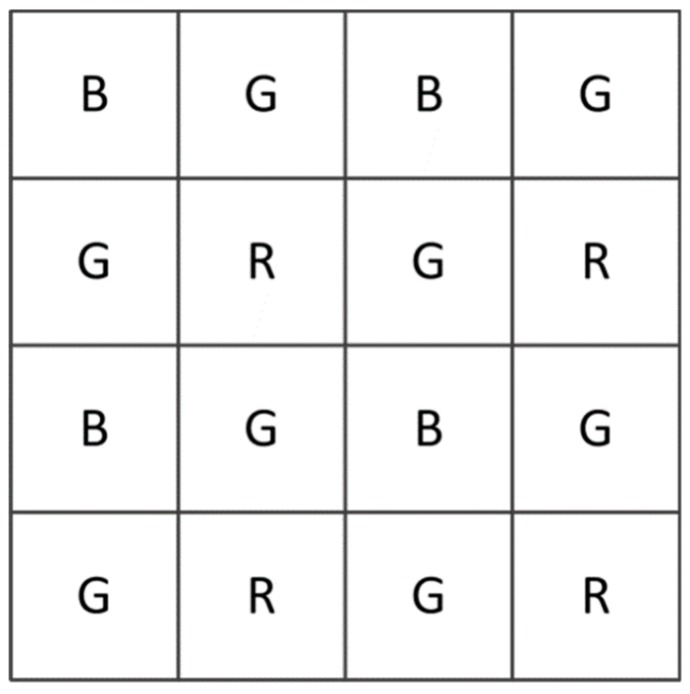
The Bayer arrangement of color filters.

The PSF measurement can be affected by a demosaicing algorithm for color filter arrays (CFAs) and sensor noise. PSFs are usually not large, and their size can even be 3 × 3 pixels, if the field of view (FOV) is well focused. The Bayer filter array [6] is a CFA that is used in most single-chip digital image sensors. As shown in Figure 1, the Bayer filter pattern is 25% red, 25% blue and 50% green, which means that 75% red, 75% blue and 50% green information are estimated using the demosaicing algorithm [7,8]. This will produce a significant error in the PSF measurement.

**Figure 2:**
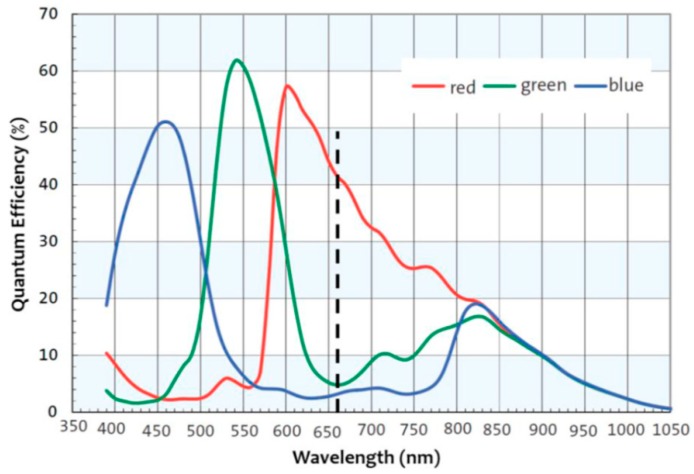
Spectral response of the three different color filters of a commercially available color image sensor array, the Aptina MT9M034. The cutoff frequency of an IR-cut filter that is typically employed in cameras is shown with the dashed line.

**Figure 3:**
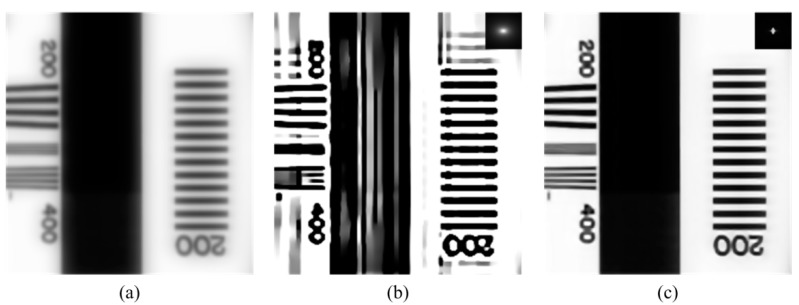
(**a**) Image blurred by 532-nm (point spread function) PSF; (**b**) image restored from (**a**) using 450-nm PSF. The 450-nm PSF is shown on the top right; (**c**) image restored from (**a**) using 532-nm PSF. The 532-nm PSF is shown on the top right.

Furthermore, color filters of real image sensors are wide-band, and PSFs vary with the wavelength [9]. Because the objects have various spectra, the PSFs of the image received by sensors are not fixed. Measuring PSFs directly can lead to their misuse. The spectral response of a color image sensor is shown in Figure 2 [10]. The blue channel can even receive 532-nm light. Assuming that the spectrum of the object is mostly 532-nm light, the blur in the blue channel would be caused by 532-nm PSF. If the 450-nm PSF is used as the PSF of the blue channel in deconvolution to reduce the blur, the misuse of the PSF will lead to a poor restored result, as shown in Figure 3.

Blind deconvolution estimates PSFs from blurred images directly. In principle, blind estimation will not have the problem of misusing PSFs owing to wide-band sensors. However, the blind estimation of PSFs relies heavily on the information in the blurred image. The PSF would be wrongly estimated if the information in the FOV of the optical system is not adequate. Moreover, blind deconvolution, which estimates blur and image simultaneously, is a strongly ill-posed problem, and can be unreliable as a result.

**Figure 4:**
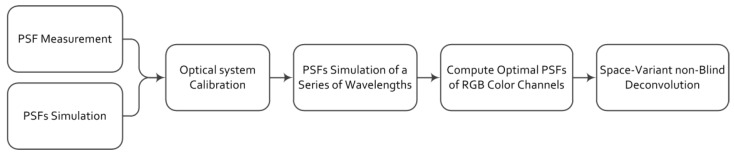
Flowchart of our proposed method.

We present an optimal PSF estimation method based on PSF measurements. Narrow-band PSF measurements and image-matching technology are used to calibrate the optical system, which makes it possible to simulate a real lens. Then, we simulate the PSFs in the wavelength pass range of each color channel all over the FOV. The optimal PSFs are computed according to these simulated PSFs, which can reduce the blur caused by the PSFs of every wavelength in the channel without introducing severe artifacts. A flowchart of the method is illustrated in Figure 4.

By utilizing the PSF measurements, our proposed method can obtain the PSFs of arbitrary wavelengths all around the FOV accurately, and can reduce the interference of noise and the demosaicing algorithm. The use of optimal PSFs avoids the artifacts caused by the misuse of PSFs owing to the use of wide-band sensors, which makes the results of image restoration robust.

The paper is organized as follows. The related works are presented in Section 2. In Section 3, the optical system calibration based on PSF measurements is described. Section 4 demonstrates the estimation of optimal PSFs. We show the results of optical system calibration and the restored images from real-world images captured by our simple optical system in Section 5. Finally, the conclusions are presented in Section 6.

## 2. Related Work

Many recent works have been proposed to estimate and remove blur due to camera shake or motion [11,12,13,14,15]. Most of them used blind estimation methods based on blurred images. However, optical blur has received less attention.

According to the properties of optical blur, some works introduced priors or assumptions in the blind method to estimate optical blur kernel [16,17,18,19]. However, the optical blur is spatially varying, so some fields of the blurred image may not have sufficient information to estimate the PSFs.

Some works created calibration boards. After obtaining blurred images and clear images of the calibration boards, PSFs were computed using deconvolution methods [3,20,21,22]. Further, Kee et al. [22] developed two models of optical blur to fit the PSFs, so that the models can predict spatially varying PSFs.

The most closely related work is [23]. Instead of estimating PSFs from blurred images, Shih et al. used measured PSFs to calibrate lens prescription, and computed fitted PSFs by simulation. However, real sensors are wide-band. The misuse of PSFs had not been considered in this work.

Kernel fusion [24] looks like the optimal PSF computation of our work, but it is actually different. Kernel fusion fused several blind kernels estimated by other papers and generated a better one as a result. In our work, the real kernel is a linear combination of multiple PSFs with unknown proportions. We compute an optimal PSF which can handle blurs caused by all these multiple PSFs stably.

## 3. PSF Measurement and Optical System Calibration

Real optical systems differ from designed optical systems owing to manufacturing tolerances and fabrication errors. Thus, the optical system needs to be calibrated before PSF simulation. Fortunately, for a simple optical system, the PSF errors caused by tolerances are very small after sensor-plane compensation. We performed many times of tolerance disturbances randomly within a reasonable range on the designed optical system using CODE V optical design software, including the radius tolerance, thickness tolerance, element decentration tolerance, element tilt tolerance and refractive index tolerance. After the sensor-plane compensation, the changes of the PSFs are much smaller than the errors introduced by the assumption that there is a constant PSF in each patch in spatially varying deconvolution. Thus, we replaced the optical system calibration with the sensor-plane calibration.

PSF measurements and image-matching technology are used in sensor-plane calibration. We compute the match between measured PSFs and simulated PSFs of different sensor-plane positions. The most matching position is the sensor-plane position after sensor-plane compensation.

**Figure 5:**
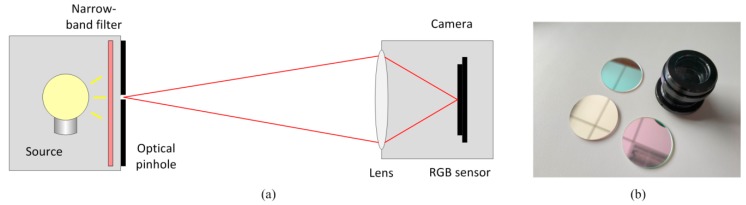
(**a**) Experimental setup of PSF measurement; and (**b**) the self-designed lens and three narrow-band filters.

The PSF measurement setup is shown in Figure 5a, and consists of a self-designed two-lens optical system with a Sony IMX185LQJ-C image sensor, an optical pinhole, light-emitting diode (LED) lamps, and three narrow-band filters whose pass ranges are around 650 nm, 532 nm and 450 nm. The LED lamp, the optical pinhole, and the narrow-band filter are installed in a black box.

The measurements only need to be done for a single object plane. Once the sensor-plane position is calibrated, we can simulate the PSFs of any object plane. We first adjust the position of the sensor to make the image clear and fix it. Then, the measured PSF can be captured by the camera. We measure the PSFs of different FOVs by moving the black box perpendicular to the optical axis. The exposure time is controlled to avoid the saturation of the light intensity.

The above experiment is used to measure the different field PSFs of 650 nm, 532 nm and 450 nm in a fixed and unknown sensor-plane position. Then, we simulate different-field PSFs of 650 nm, 532 nm and 450 nm in different sensor-plane positions using CODE V to compute the match with the measured PSFs.

**Figure 6:**
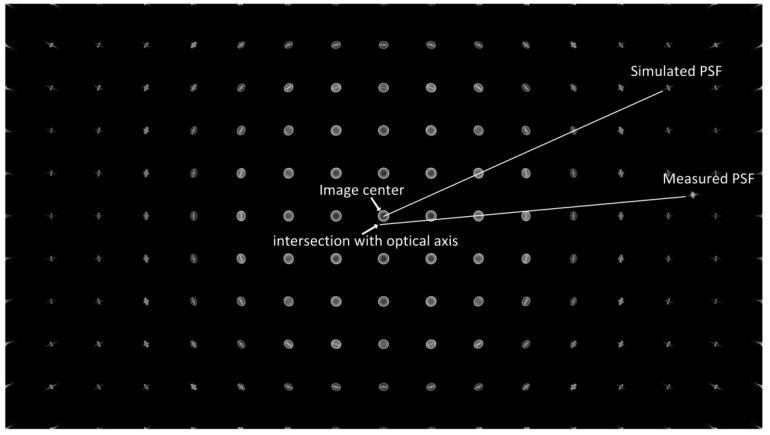
Diagram of PSF field matching.

Before the sensor-plane matching, we need to find the simulated PSF that is in the same field as the measured PSF. As show in Figure 6, the PSFs array is a set of spatially varying simulated PSFs of 650 nm in one sensor-plane position. The single PSF out of the array is a measured PSF of one field. Optical systems have rotational symmetry with respect to the optical axis. In the simulation, the intersection of optical axis and the sensor is the image center. However, in the real optical system, the intersection will deviate from the image center. According to the rotational symmetry of the optical system, if the distance from the real intersection to the measured PSF is close to the distance from the image center to the simulated PSF, their fields match.

We first calibrate the realistic intersection using the least-square method. In optical systems, a PSF that does not lie on the optical axis of the lens has reflection symmetry with respect to the meridional plane. The symmetric axis of the PSF goes through the intersection of the optical axis and the sensor. We find these symmetric axes of the measured PSFs. Considering the error, the point with the least sum of distances from these symmetry axes is the best choice of the intersection.

The distances from simulated PSFs to the image center form a set S. We find the element in S closest to the distance from the intersection of the optical axis and the sensor to the measured PSF. If their difference is less than Δd which represents the acceptable field error, the simulated PSF and the measured PSF are considered to be in the same field. If their difference is more than Δd, which means that there is no simulated PSF in the same field as the measured PSF, the measured PSF will be abandoned. After field matching, we rotate the simulated PSFs in different sensor planes to make them have the same tilt angle as the measured PSF.

The measured PSFs are affected by a demosaicing algorithm and sensor noise, but their sizes and shapes are hardly affected. Therefore, a template matching approach can be used to find the real sensor plane. We compute the maximum of the normalized cross-correlation matrix of the simulated PSFs and the measured PSF as the matching degree [25]. The sensor plane in which the matching degree of the simulated PSF and the measured PSF is highest is the calibration result. The normalized cross-correlation matrix is expressed as follows:

(1) $$\gamma (x,y)=\frac{\sum_{s}\sum_{t}\left[w(s,t)-\bar{w}\right]\left[f(x+s,y+t)-{\bar{f}}_{xy}\right]}{{\left\{\sum_{s}\sum_{t}{\left[w(s,t)-\bar{w}\right]}^{2}\sum_{s}\sum_{t}{\left[f(x+s,y+t)-{\bar{f}}_{xy}\right]}^{2}\right\}}^{\frac{1}{2}}},$$

where *w* is the measured PSF, and *f* is the simulated PSF. $\bar{w}$ is the average value of *w*, and ${\bar{f}}_{xy}$ is the average value of *f* in the overlapping region of *w* and *f*.

There is a potential ambiguity that more than one sensor plane has a high matching degree. However, for the best sensor plane, the matching degrees are high in all fields. We calculate the average of the matching degrees in all fields to prevent ambiguity.

## 4. Optimal Point Spread Function Estimation

After calibration, the PSFs of every wavelength in all fields can be simulated accurately. However, real sensors are wide-band. Each color channel of a real sensor allows light in a large wavelength range to pass through. In the daily use of cameras, the complex illumination and the multiple reflectance of objects make it difficult to obtain the spectrum received by sensors. Thus, it is difficult to calculate the real PSF by weighting. We estimate an optimal PSF according to the simulated PSFs in the wavelength pass range of the color channel, which can reduce the blur caused by each wavelength PSF in the channel without introducing severe artifacts.

In optical systems, the latent sharp image *i* is blurred by PSF $k_{\lambda }$. The observed image *b* can be expressed as follows:

(2) $$b=i*k_{\lambda }+n,$$

where ∗ is the convolution operator, and *n* is the additive noise. The effect of noise can be suppressed significantly by employing regularization in state-of-the-art deconvolution methods, so we will not consider noise in the following discussion.

In the frequency domain, Equation (2) can be written as follows:

(3) $$B=I\times K_{\lambda },$$

where the terms in capital letters are the Fourier transforms of the corresponding terms in Equation (2).

If we use a new PSF *k_o_* in the deconvolution to restore the image, the new resultant image *i*′ in the frequency domain can be expressed as follows:

(4) $$I^{\prime }=I\times \frac{K_{\lambda }}{K_{o}}.$$

Therefore, the new resultant image can be seen as the latent sharp image blurred by $F^{-1}\left(K_{\lambda }/K_{o}\right)$, where $F^{-1}$ represents the inverse Fourier transform.

In optical systems, the Fourier transform of the PSF is the optical transfer function (OTF), which can be considered as a combination of the modulation transfer function (MTF) and the phase transfer function (PTF):

(5) $$OTF=MTF\times e^{iPTF}.$$

Therefore, the equivalent OTF of the optical system after deconvolution is as follows:

(6) $$OTF_{e}=\frac{K_{\lambda }}{K_{o}}=\frac{MTF_{\lambda }}{MTF_{o}}e^{i(PTF_{\lambda }-PTF_{o})},$$

where $MTF_{\lambda }$ and $PTF_{\lambda }$ are the MTF and PTF of $k_{\lambda }$, respectively; *MTF_o_* and *PTF_o_* are the MTF and PTF of *k_o_*, respectively. The equivalent MTF and PTF of the optical system after deconvolution are as follows:

(7) $$\begin{array}{l} MTF_{e}=\frac{MTF_{\lambda }}{MTF_{o}}\\ PTF_{e}=PTF_{\lambda }-PTF_{o} \end{array}$$

MTF is related to contrast reduction. Ideally, MTF is a constant 1, which means that the modulation of the image is the same as the modulation of the object. Actually, some of the information of the object will be lost in a real optical system, and MTF will be less than 1. If MTF is more than 1, the modulation will increase, and the intensity of the pixels will be saturated easily. As shown in Figure 3b, this saturation is unacceptable. Thus, for an image that is blurred by a series of PSFs, that is, $k_{\lambda_{1}}$, $k_{\lambda_{2}}$, …, $k_{\lambda_{n}}$, *MTF_e_* should be close to 1 and should not exceed 1. The *MTF_o_* should be as follows:

(8) $$MTF_{o}(\nu )=\max({\left\{MTF_{\lambda_{i}}(\nu )\right\}}_{i=1\ldots n}),$$

where ν is the spatial frequency, and $MTF_{\lambda_{i}}$ represents the MTF of $k_{\lambda_{i}}$.

PTF indicates the shape and size information of PSF. Ideal PSF is spot-like or disk-like. The energy is concentrated, and the PTF is generally a constant 0. For a set of PSFs, there are various shapes and sizes. The PTFs are close at some spatial frequency area and have a large difference at other spatial frequency areas. In the close area, we calculate the average of these PTFs as the PTF of the optimal PSF to make $PTF_{e}(\nu )$ close to 0 for this entire set of PSFs. In the other area, there is no fixed value of PTF to restore $PTF_{\lambda }$ of all the wavelengths in the pass range of the color channel. We set the $PTF_{o}$ to 0 in the area to avoid the presence of artifacts caused by incorrect recovery, although the blur caused by $PTF_{\lambda }$ in the spatial frequency area will remain.

The optimal PSF *k_o_* can be expressed as follows:

(9) $$k_{o}=F^{-1}\left(MTF_{o}e^{iPTF_{o}}\right).$$

## 5. Result

In this section, we show the results of the optical system calibration, and we compare the restored results from real-world images captured by our simple optical system.

**Figure 7:**
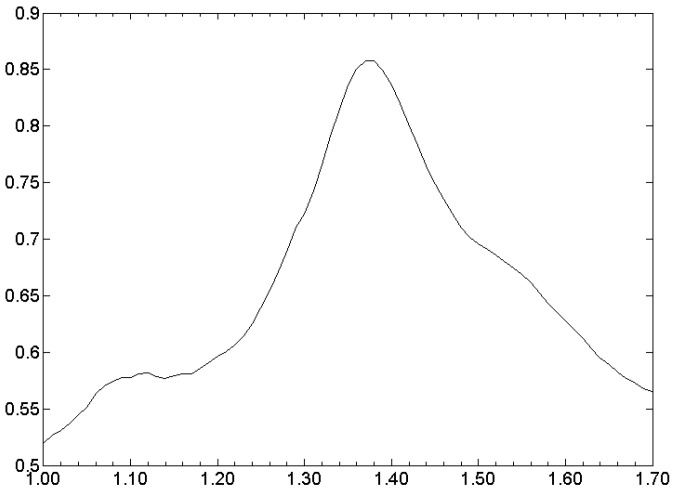
Sensor-plane matching curve. The abscissa is the distance from the simulated sensor plane to the designed sensor plane, and the units are in mm. The ordinate is the matching degree of the simulated PSFs and the measured PSF.

**Figure 8:**
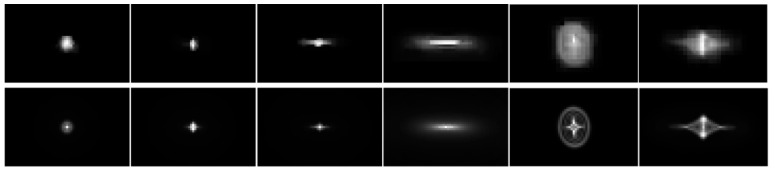
Comparison of the measured PSFs and the matched simulated PSFs. The measured PSFs are on the top, and the matched simulated PSFs are on the bottom.

The sensor-plane matching curve is shown in Figure 7. Where the abscissa is 1.38 mm, the matching degree is the highest which is 0.858. Note that the upper limit of the matching degree is 1. This means that the matched simulated PSFs are very similar to the measured PSFs. Figure 8 shows the comparison of the measured PSFs and the matched simulated PSFs. The sizes and shapes of the matched simulated PSFs in all fields are close to the measured PSFs. We can see that the simulated PSFs have more details and are more accurate, because they are not affected by noise or demosaicing.

We simulated the PSFs for which the wavelengths range from 450 nm to 650 nm using CODE V, and the interval is 10 nm. According to the spectral sensitivity characteristics of the Sony IMX185LQJ-C image sensor, the wavelength pass ranges of the color channels are extracted. The pass ranges of the red channel, the green channel and the blue channel are 570–650 nm, 470–640 nm and 450–530 nm, respectively. After computing the optimal PSFs, the blurred image is divided into 7 × 13 rectangular overlapping patches and restored using the deconvolution method in [26].

For comparison, we also restored the patches of the image using blind-estimated PSFs [13] and selected wavelength PSFs. The deconvolution method that was employed was the same as in our proposed method, and all the deconvolution parameters were the same. The selected wavelengths are 620 nm, 530 nm and 470 nm, which respectively have the highest spectral sensitivities in the red channel, the green channel and the blue channel of the Sony IMX185LQJ-C image sensor.

**Figure 9:**
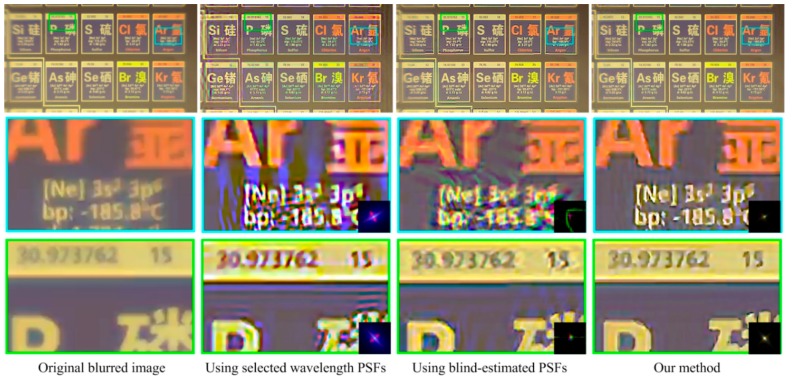
Results for the periodic table image. The top-left image is the captured blurred image. The right three images on the top row are the restored results using three sets of spatially varying PSFs. The bottom two rows are the details of the top-row images. The bottom-right corners of the detail images of the restored results show the used local PSFs.

**Figure 10:**
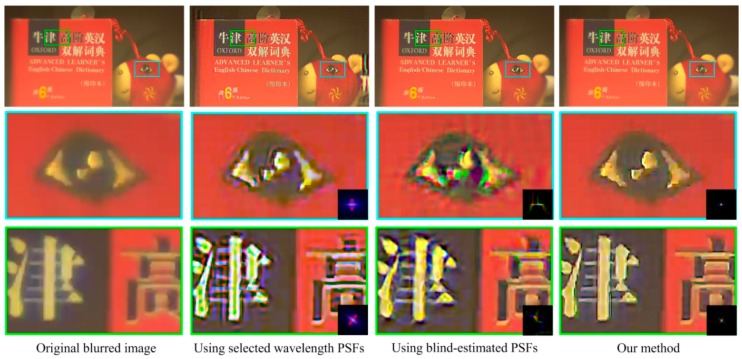
Results for the dictionary image. The top-left image is the captured blurred image. The right three images on the top row are the restored results using three sets of spatially varying PSFs. The bottom two rows are the details of the top-row images. The bottom-right corners of the detail images of the restored results show the used local PSFs.

**Figure 11:**
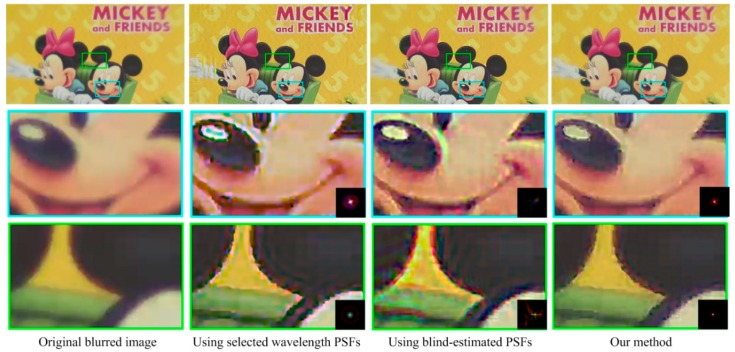
Results for the Mickey image. The top-left image is the captured blurred image. The right three images on the top row are the restored results using three sets of spatially varying PSFs. The bottom two rows are the details of the top-row images. The bottom-right corners of the detail images of the restored results show the used local PSFs.

Figure 9, Figure 10 and Figure 11 show the restored results obtained from real-world images captured by our simple optical system. The resolution is 1920 × 1080 pixels. The results obtained using selected wavelength PSFs and blind-estimated PSFs include noticeable artifacts. They can hardly handle images reasonably that are captured by our simple optical system with a wide-band sensor. Compared to the other methods, our proposed method has fewer artifacts and the image quality is more stable. Furthermore, our method is several-dozen times faster than the blind method [13], because we only need simple calculations to estimate the PSFs.

We have not compared our results with the restored image using measured PSFs because it is difficult and time consuming to measure PSFs in all fields accurately. As shown in Figure 8, the measured PSFs are significantly affected by noise and demosaicing. The restored results will be worse than the results obtained using selected wavelength PSFs.

## 6. Conclusions

In this paper, we present an optimal PSF estimation method that is based on PSF measurements. By performing PSF measurements and PSF simulations, the proposed method can obtain PSFs more accurately. Considering the use of wide-band sensors, the method can restore images of simple optical systems stably without severe artifacts. The experiment carried out using the real-world images captured by a self-designed two-lens optical system shows the effectiveness of our method.

In the optical system calibration part, the proposed method ignores the error caused by residual tolerance after sensor-plane compensation. The method is suitable for lenses with high machining accuracy. However, for low-precision lenses, there is a need for a more precise optical system calibration method.
